# Identifying Disparities in Timely Receipt of Radiation After Breast‐Conserving Surgery

**DOI:** 10.1155/tbj/9942451

**Published:** 2026-02-02

**Authors:** Nicole Reyes, Camila Ortega, Amanda Mendiola, Mary Murray, Andrew Fenton, Adina Brett-Morris, Caroline Mangira

**Affiliations:** ^1^ Department of Surgery, Cleveland Clinic Akron General, 1 Akron General Ave, Akron, 44307, Ohio, USA, akrongeneral.org; ^2^ Department of Health Sciences, Cleveland Clinic Akron General, 1 Akron General Ave, Akron, 44307, Ohio, USA, akrongeneral.org; ^3^ Department of Research, Cleveland Clinic Akron General, 1 Akron General Ave, Akron, 44307, Ohio, USA, akrongeneral.org

## Abstract

**Introduction:**

Radiation therapy after breast‐conserving surgery reduces local recurrence and improves survival. The new standard set forth by the Commission on Cancer (CoC) requires that radiation be initiated in less than or equal to 60 days of definitive surgery for patients receiving breast‐conserving surgery for Stages I–III breast cancer who do not undergo adjuvant chemo or immunotherapy. Timely access to radiation is critical, and yet there still exists a modest number of patients who experience delays in the initiation of radiation. We aim to highlight this disparity at our institution and identify the socioeconomic factors that contribute to it.

**Methods:**

Using the Breast Cancer Registry, we conducted a retrospective analysis of women diagnosed with Stages I–III breast cancer, who underwent breast‐conserving surgery between 2011 and 2021. Women who received chemotherapy were excluded. We stratified patients based on socioeconomic and other factors and examined which factors attributed to an increased interval from surgery to initiation of radiation greater than the current standard of 60 days.

**Results:**

A cohort of 427 women meeting the inclusion criteria was identified. Most patients received adjuvant radiation within the new standard of 60 days from definitive surgery (72.4%). However, patients of White race were significantly more likely to receive adjuvant radiation within 60 days of final surgery (74.7%) compared to patients of Black race (55.6%). In addition, patients with private insurance or Medicare were more likely to receive adjuvant radiation within the current set standard (74.9% and 74.5%, respectively) in comparison to patients with Medicaid (50.0%).

**Conclusion:**

This analysis identifies disparities in breast cancer treatment among minority populations at our institution. It also suggests that insurance status can affect the receipt of treatment in a recommended time frame. There is research that shows a delay in radiation impairs survival. These results indicate that improving access to timely adjuvant radiation may be leveraged to lessen disparities experienced by minority races regardless of insurance status.

## 1. Introduction

Breast conservation therapy has been widely accepted as the standard of care in the treatment of breast cancer patients for decades [1]. A small subset of patients has been identified as appropriate for omitting adjuvant radiation [2]; however, adjuvant radiation is generally considered standard for those with invasive breast cancer undergoing breast‐conserving surgery.

The addition of adjuvant radiation with breast‐conserving surgery has been shown to reduce rates of locoregional recurrence and improve survival: A meta‐analysis of several randomized trials of lumpectomy with or without adjuvant radiotherapy showed that radiation significantly reduced the likelihood of in‐breast recurrence by 19% at 5 years and also improved breast cancer‐specific mortality by 5% at 15 years [3].

The Commission on Cancer (CoC) recently updated the quality measure regarding the timing of radiotherapy following breast‐conserving surgery. Previously, the acceptable interval time from invasive breast cancer diagnosis to the start of radiotherapy was 1 year. This was to ensure enough time for those undergoing systemic therapy that radiation was not omitted. However, a recent study demonstrated that even in patients not undergoing chemotherapy, up to 25% have radiation delays from surgery greater than 60 days, and this resulted in significant increases in the hazard of mortality [4]. The new standard set forth by the CoC now requires that radiation, when administered, be initiated in less than or equal to 60 days of definitive surgery for patients receiving breast‐conserving surgery for Stages I–III breast cancer who do not undergo adjuvant chemo or immunotherapy [5].

Despite abundant research identifying the importance of timely receipt of radiation in breast cancer care, unfortunately, delays still exist [6]. The National Cancer Institute (NCI) defines cancer health disparities as “adverse differences between certain groups in cancer measures such as incidence, prevalence, morbidity, mortality, survivorship, screening rates, and quality of life.” NCI suggests certain population groups are at higher risk of experiencing these disparities, including groups characterized by race, ethnicity, gender identity, geographic location, income, education, age, sexual orientation, and others [7]. Although there appear to be promising trends reflecting a decline in disparities with breast cancer treatment in recent years, barriers to treatment still persist when considering race and other socioeconomic factors [8, 9]. We aim to highlight this disparity at our institution and identify the population groups and socioeconomic factors that may contribute to this.

## 2. Materials and Methods

This retrospective analysis was conducted using data gathered from the Akron General Breast Cancer Registry between 2011 and 2021. The project was submitted to the Institutional Research Review Board where it was decided that it met the criteria for Quality Assurance/Improvement thus not requiring IRB oversight. The cohort included women diagnosed with nonmetastatic invasive breast cancer (Stages I–III) ranging from 18 to 69 years of age. Staging was derived from the AJCC staging. All patients included in the cohort underwent breast‐conserving surgery, which included recommendations for adjuvant radiation. Women who received chemotherapy were excluded given the updated dichotomized quality measure, which allows women undergoing systemic therapy a time interval to receive adjuvant radiation of 1 year.

Our primary dependent variable of interest was initiation of adjuvant radiation within the newly accepted time interval, now defined as < 60 days from definitive surgery. We then stratified patients based on socioeconomic and other factors and examined which attributed to an increased time interval from surgery to initiation of radiation greater than the current standard of 60 days.

Population groups at risk of experiencing cancer healthcare disparities are groups based on race, income, and health insurance status, which we used as our independent variables. We hypothesized that identifying and studying these variables could lend themselves to actionable and targeted options for interventions to help address inequalities in the context of breast cancer treatment and patient outcomes.

Race and primary payor at diagnosis were both collected at the time of the registry. Data on race were segmented into three subsets: White, Black, and Other (which included race not otherwise specified [NOS]). Primary payer at diagnosis was categorized into data subsets for Insurance NOS, Medicare, Medicaid, not insured, or private insurance. Zip code data was available in the Breast Cancer Registry, and we used this information to define distance to the radiation oncology center: ≤ 10 miles, 10–30 miles, and > 30 miles. Because data on individual finances were not collected, we used the most current Census data based on Zip Codes (2020) to assign a mean household income to each of the patients. Based on the distributions of household income in the registry, we defined “low” income as < $62,186 and “high” income as > $74,323.

Statistics are presented as frequencies and percentages, and comparisons between groups are obtained using chi‐squared tests or Fisher’s exact tests as appropriate. A significance level of 0.05 was assumed for all tests. Analyses were performed using SAS Software (Version 9.4, Cary, NC).

## 3. Results

A cohort of 427 women diagnosed with Stages I–III breast cancer between 2011 and 2021 who underwent breast‐conservation surgery was identified. All underwent adjuvant radiotherapy and none received chemotherapy. The median age was 58. Most women were White (*n* = 383, 89.9%), and Black women comprised 8% (*n* = 36) of the cohort. Most women were married (*n* = 272, 65.2%) and had private insurance (*n* = 239, 59.7%). 309 (72.4%) patients started radiation treatment within 60 days of definitive surgery, leaving 118 (27.6%) patients who had a delay in starting radiation of > 60 days from her final surgery.

The patient characteristics that were analyzed in the study are identified in Table 1, including the number of patients who received radiation within 60 days.

**TABLE 1:** Patient characteristics (*n* = 427).

Age	58.87 (median)
Race	No. (%)
White	383 (89.91)
Black	36 (8.45)
Other	7 (1.64)
Marital status at diagnosis	
Divorced	62 (14.87)
Married	272 (65.23)
Single	64 (15.35)
Widowed	19 (4.56)
Primary payer at diagnosis	
Insurance NOS^∗^	17 (4.25)
Medicare	106 (26.50)
Medicaid	32 (8.00)
Not insured	6 (1.50)
Private	239 (59.75)
Household income	
≤ 62,186	152 (35.76)
62,187–73,504	58 (13.65)
73,505–74,323	114 (26.82)
> 74,323	101 (23.76)
Radiation within 60 days of definitive surgery?	
No	118 (27.63)
Yes	309 (72.37)

^∗^NOS = not otherwise specified.

Significant racial differences were found regarding the receipt of radiation within 60 days from surgery. Patients of White race (74.7%) were significantly more likely to receive adjuvant radiation within 60 days from surgery as compared to patients of Black race (55.6%) or other (28.6%) (*p* = 0.0017) (Table 2).

**TABLE 2:** Impact of race, insurance, household income, and distance on receiving radiation within 60 days of definitive surgery (n = 427).

Characteristic	Radiation within 60 days of definitive surgery		*p* value
	Yes (*n* = 309) no. (%)	No (*n* = 118) no. (%)	
Race			0.0017
White	286 (74.67)	97 (25.33)	
Black	20 (55.56)	16 (44.44)	
Other	2 (28.57)	5 (71.43)	
Primary payer at diagnosis			0.0625
Insurance NOS^∗^	12 (70.59)	5 (29.41)	
Medicare	79 (74.53)	27 (25.47)	
Medicaid	16 (50.00)	16 (50.00)	
Not insured	5 (83.33)	1 (16.67)	
Private	179 (74.90)	60 (25.10)	
Household income			0.6345
≤ 62,186	111 (73.03)	41 (26.97)	
62,187–73,504	38 (65.52)	20 (34.48)	
73,505–74,323	85 (74.56)	29 (25.44)	
> 74,323	74 (73.27)	27 (26.73)	
Distance to the radiation oncology center (miles)			0.5841
≤ 10	150 (71.43)	60 (28.57)	
10–30	141 (72.31)	54 (27.69)	
> 30	18 (81.82)	4 (18.18)	

^∗^NOS = not otherwise specified.

In addition, patients with private insurance or Medicare were more likely to receive adjuvant radiation within the current set standard (74.9% and 74.5%, respectively) in comparison to patients with Medicaid (50%), although this was not deemed to be statistically significant (*p* = 0.0625). There was also a small subset of patients who were not insured (*n* = 6), and the majority of those patients did receive radiation within the appropriate timeframe (83.3%) (Table 2).

Differences in household income were not found to be associated with the timely receipt of radiation within 60 days. Patients in the lowest income group were almost as likely to receive radiation in the 60 days from surgery as patients in the highest income group (73.0% and 73.2%, respectively) (Table 2). Likewise, the distance to the radiation oncology center was not independently associated with the receipt of timely radiation; patients traveling more than 30 miles were as likely to receive radiotherapy within 60 days as patients living closer to our institution.

## 4. Discussion

In our review of 427 women < the age of 70 who underwent breast‐conserving surgery including adjuvant radiation for Stages I–III breast cancer, women of White race were significantly more likely than women of Black race to receive adjuvant radiation in the new quality standard time of < 60 days from definitive surgery (if not undergoing systemic therapy).

A recent review demonstrated that still about 25% of patients undergoing breast‐conserving surgery without chemotherapy have a delay in the initiation of radiation of > 8 weeks from the definitive surgery, which is associated with impaired survival [4]. Similar to those findings, approximately 27.6% of our patient cohort encountered a similar delay in radiation of > 60 days from final surgery. This highlights that delays in timely treatment of radiation are present even without potentially exacerbating factors such as the socioeconomic and racial factors that we set out to analyze.

Even though our findings did not reach significance, a trend still existed in which patients with private insurance or Medicare were more likely to be compliant with the new standard radiation time interval than other patients with Medicaid, which is similar to other studies [10]. Furthermore, larger distances were not associated with differences in the timing of adjuvant radiation, as seen in previous publications [11], which indicates that distance is less of a barrier than other sociodemographic factors.

The findings in this study may be constrained by some limiting factors. These limiting factors may include a small sample size, which was drawn from patients at a single institution, such that the findings are not generalized and may not apply to a broader population. Also, these findings do not account for other factors such as personal choice or patient compliance (or noncompliance), both of which could impact outcomes and interpretations of the analysis. Therefore, the results underscore the need for additional research at other institutions.

Given the recent update of the quality metric standard, receipt of radiation within 60 days or less from definitive surgery in BCT in patients not undergoing systemic therapy, there are few studies analyzing this time interval. Not only does our analysis identify a disparity in breast cancer treatment among minority populations at our institution, but we have also discovered that approximately 27% of patients would not have met the current standard for timely receipt of radiation, which may have an impact on overall survival. These results indicate that improving access to timely adjuvant radiation may be leveraged to lessen disparities experienced by minority races regardless of insurance status.

## 5. Conclusion

This analysis identifies a disparity in breast cancer treatment, specifically regarding receipt of adjuvant radiation as part of breast‐conservation therapy. These findings collectively add to our main goal of identifying and raising awareness of health disparities within our community so that we may take meaningful steps toward addressing them and achieving equity. It is paramount to identify the patients at risk of delays in breast cancer care and to constantly engage our most vulnerable communities by promoting safe environments where patients feel included, and barriers of care are recognized and overcome.

## Funding

No funding was received for this study.

## Disclosure

These findings have been previously presented at the American Society of Breast Surgeons 25^th^ Annual Meeting Poster Session on April 12, 2024.

## Conflicts of Interest

The authors declare no conflicts of interest.

## Data Availability

The data that support the findings of this study are available from the Cleveland Clinic. Restrictions apply to the availability of these data, which were used under license for this study. Data are available from the authors with the permission of the Cleveland Clinic.
